# Coiled-Coils: The Molecular Zippers that Self-Assemble Protein Nanostructures

**DOI:** 10.3390/ijms21103584

**Published:** 2020-05-19

**Authors:** Won Min Park

**Affiliations:** Tim Taylor Department of Chemical Engineering, Kansas State University, Manhattan, KS 66506, USA; wmpark@ksu.edu; Tel.: +1-785-532-5597

**Keywords:** coiled-coils, nanostructures, protein nanotechnology, protein origami, self-assembly

## Abstract

Coiled-coils, the bundles of intertwined helical protein motifs, have drawn much attention as versatile molecular toolkits. Because of programmable interaction specificity and affinity as well as well-established sequence-to-structure relationships, coiled-coils have been used as subunits that self-assemble various molecular complexes in a range of fields. In this review, I describe recent advances in the field of protein nanotechnology, with a focus on programming assembly of protein nanostructures using coiled-coil modules. Modular design approaches to converting the helical motifs into self-assembling building blocks are described, followed by a discussion on the molecular basis and principles underlying the modular designs. This review also provides a summary of recently developed nanostructures with a variety of structural features, which are in categories of unbounded nanostructures, discrete nanoparticles, and well-defined origami nanostructures. Challenges existing in current design strategies, as well as desired improvements for controls over material properties and functionalities for applications, are also provided.

## 1. Introduction

**Figure 1:**
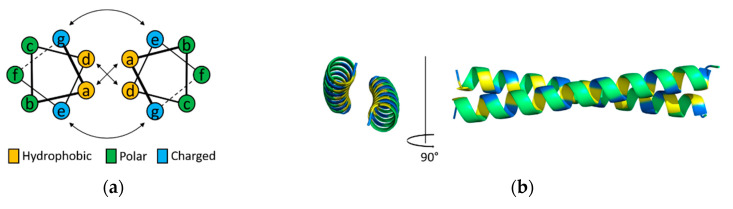
Structure and interface of a dimeric coiled-coil; (**a**) A helical wheel diagram that presents amino acid residues located at the characteristic positions labeled *abcdefg*. Their interactions at the interface are indicated by arrows; (**b**) Top (left) and side view images (right) of the backbone structure. The structural model was generated using CCBuilder 2.0 [11].

Coiled-coils are α-helical structural protein motifs that wrap each other into supercoiled helices. They play essential roles as subunits that oligomerize protein complexes involved in biological processes or that form structural elements of biological materials [1]. For example, the basic leucine-zipper proteins, a class of transcription factors, contain coiled-coil motifs that mediate dimerization [2], which influences biochemical functions such as gene regulation in diverse species [3]. Tropomyosin, a regulator of the actin cytoskeleton, polymerizes along actin filaments [4], and the coiled-coil stiffness and stability are associated with muscle functioning [5]. Beyond such essential biological roles of coiled-coils, understanding their molecular basis has been sought over the past three decades. The sequences, structures, and interactions of coiled-coils have been studied extensively and well documented in many reviews [6,7,8,9,10]. In brief, the sequence-to-structure relationships are described that hydrophobic and polar amino acid residues are patterned in a repeat of seven residues (Figure 1). The heptad repeats of coiled-coils are labeled *abcdefg*, which are indicated in a helical wheel diagram that denotes the top view of helices. A helical diagram of a typical dimeric coiled-coil shows that hydrophobic amino acid residues are placed in the positions of *a* and *d*, while polar residues are in the positions of *e* and *g*. The hydrophobic-polar patterns of amino acid residues at the interface between coiled-coils directly influence the interaction between the helices. The number of helices of a coiled-coil (e.g., dimeric, trimeric, or tetrameric) is decided by packing in the hydrophobic core at the positions of *a* and *d*. 

In addition to this well-understood molecular basis, expanded availability of experimental datasets has facilitated methodological advancements for the de novo design of synthetic coiled-coil sequences. Rational [12,13,14], computational [15,16,17,18,19,20], or combined approaches [21] have been used to design highly versatile coiled-coil toolkits. For example, synthetic coiled-coils with controlled binding affinity [22,23], specificity [10,15,16,17], helix orientation [18], and oligomeric state [14,21] have been designed. Such controls have significantly improved the utility of coiled-coils as tags or subunits that directed self-assembly of engineered molecular complexes, which modulate physical and biological processes in a range of applications. Recent reviews summarize applications of coiled-coils in broad fields, which include synthetic biology [24], medicine [25], and material sciences [9]. 

This review focuses on the bottom-up creation of supramolecular nanostructures using coiled-coils. As self-assembling modules, coiled-coils mediate connections between protein building blocks that self-assemble into higher-order nanostructures. In such systems, coiled-coils are modularly combined or modified to direct assembly into desired shapes and morphologies. The type of coiled-coil-based nanostructures ranges from unbounded fibers [26,27] and tubes [28,29] to well-defined polygons [30,31] and polyhedrons [32,33]. Whereas many reviews are well documented about the sequences, interactions, and structures of coiled-coils, the essential basis for modular design of nanostructures or related fundamentals has not been discussed much yet. In this review, I will discuss the design principles as well as the structural features found in recently developed nanostructures, which are categorized as unbounded nanostructures, discrete nanoparticles, and well-defined origami nanostructures. A discussion on challenges existing in design and perspectives on applications follows.

## 2. Modular Design Principles

The specific associations between coiled-coils in various types (e.g., homo- or heterodimeric, dimeric or oligomeric, parallel or antiparallel) have been exploited to direct assembly of protein nanostructures. Assembling diverse supramolecular nanostructures requires at least two distinct protein–protein contacts between building blocks, as discussed in a review by Yeates et al. [34]. However, a coiled-coil has only a single-type interface that mediates the formation of a helical bundle of two or up to several helices, as illustrated in Figure 1b. The outer surface or the N- or C-terminus of coiled-coils are stable. Thus, building nanostructures other than rod-like helical bundles, the intrinsic structures of coiled-coils, cannot be achieved. It requires a strategic variation of coiled-coil sequences. For converting coiled-coils into self-assembling building blocks, coiled-coils have been combined or modified in a modular fashion. In this section, I will discuss the strategies to combine or modify coiled-coils for programed coiled-coil assembly and the underlying basis for the modular design of various nanostructures.

**Figure 2:**
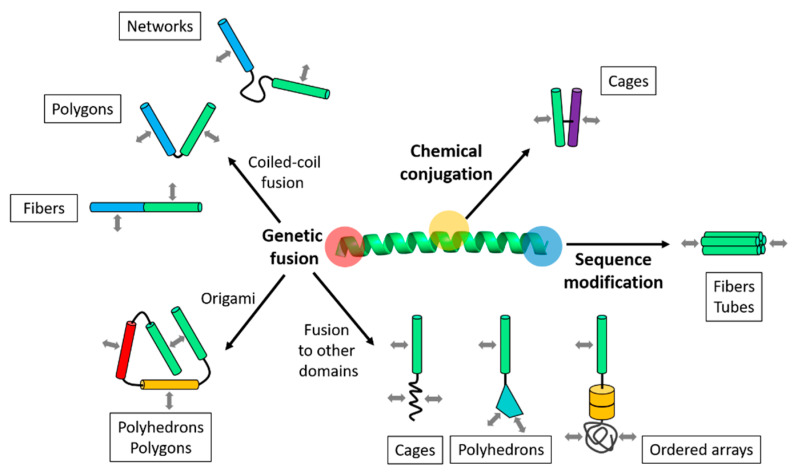
Modular design principles of coiled-coil-based protein building blocks. Building blocks are designed through variations of a coiled-coil peptide, which include genetic fusion, chemical conjugation, and sequence modification. In the genetic fusion approach, coiled-coils are linked with or without orientational controls. They are also combined into fusion proteins for origami or fused to other domains. Association contacts on each building block are indicated by arrows (gray). The types of resulting supramolecular nanostructures are indicated in the boxes, and more details are summarized in Table 1.

One of the approaches to the variation of coiled-coils is a fusion of distinct coiled-coil interfaces. When a coiled-coil forming helix is fused with other helices that have different interfaces, the resulting building block can have at least two distinct contacts (Figure 2). In the fusion, amino acid sequences of coiled-coils are put together as a single chain, so that coiled-coils are linked through their N- or C-termini. That way, a building block is designed to be connected through the introduced coiled-coil interfaces, leading to the formation of a supramolecular structure. Short linker sequences are placed between the linked coiled-coil sequences if the spacing is required. Once amino acid sequences are designed, protein building blocks are prepared by the expression of recombinant DNA [30,32] or chemical synthesis [31,35]. Recombinant expression provides advantages of high yield and flexibility in the length of fusion proteins, while chemical synthesis is fast and relatively simple but limits the production of peptides longer than about 50 amino acids. Additionally, chemical synthesis enables the incorporation of unnatural amino acid or backbone modification, which provides more opportunities for modification of coiled-coils. Chemical conjugation of synthesized coiled-coils (e.g., via disulfide bonds) is also used to combine coiled-coil interfaces into a single building block [35]. Although this approach requires additional chemical reaction and purification steps, the “side-to-side” linking of coiled-coils through the outer surfaces can be achieved. In contrast, genetic fusion only enables the “end-to-end” linking through the N- and C-termini. 

The way multiple coiled-coils are combined critically influences relative orientations of coiled-coil interfaces. It further dictates the morphologies of resulting nanostructures (Figure 2). While the type of coiled-coil interfaces (e.g., dimeric or trimeric) decides the number of chains that are bound at each contact, the angles between adjacent coiled-coil interfaces are controlled by strategic linkage of coiled-coils. For example, the angle between two heterodimeric coiled-coils was controlled approximately from 15° to 100° by the linker sequences, which decided the number of assembled subunits to be 2, 3, 4 or significantly large numbers [31]. As a result, nanostructures in various morphologies, such as polygonal shapes, fibers, or spheres, were assembled from identical pairs of coiled-coils that were flanked by linker sequences in different lengths. Intramolecular disulfide bridges between coiled-coils were also used to specify their relative orientations for self-assembly of a polyhedral nanoparticle [36]. In a different approach, chemical conjugation to the outer surfaces of trimeric and dimeric coiled-coils resulted in a wedge-shape assembly of coiled-coils. The angle between the trimers was ~10°, resulting in a curved membrane-like structure that was closed into a cage [35]. The angle can also be controlled to be ~180° by co-directional alignment. It led to the formation of fibers, where two distinct coiled-coil interfaces were linked together with no [26] or two-residue linker sequences [31], followed by longitudinal assembly.

In addition to engineering the linkage between coiled-coils, more strategies have been developed to orient coiled-coils in specific ways (Figure 2). For example, the systematic design of topologies of linked coiled-coils enabled “origami” of polygonal [30] or polyhedral nanostructures [32,33]. In this strategy, termed coiled-coil protein origami (CCPO), orientations of coiled-coils were precisely controlled by specific intra- or intermolecular dimerization of multiple distinct coiled-coils, which were arranged into the designed topologies. Other strategies include the introduction of non-covalent interactions to coiled-coils. Modification of coiled-coils with charged residues on the N- or C-termini enabled co-directional alignment of the helices [28]. The charged coiled-coils were extended in the longitudinal direction, leading to the formation of fibers and tubes. Lastly, non-coiled-coil domains were genetically fused to coiled-coils as fusion partners to orient coiled-coil interfaces. Hydrophobic peptides, symmetrically oligomerizing protein folds, or protein-polymer conjugates were combined with coiled-coils to direct the formation of polyhedral nanoparticles [37], vesicles [38], or ordered arrays [39], respectively.

Experimental validation of the modular nanostructure design is critical and done through structural characterization. Several characterization techniques have been used, depending on the structure type and resolution. Transmission electron microscopy (TEM) or scanning electron microscopy (SEM) was used to visualize nanoscale morphologies of the networks [40], fibers [27], tubes [41], cages [35,42], vesicles [38,43], and polyhedrons [36,37,44]. Also, atomic force microscopy (AFM) was used to characterize the CCPO nanostructures [30,32]. Although these techniques are not enough to provide atomic-level details, orientations of coiled-coils within the nanostructures were further confirmed by other bioassays [38,42,43,45]. Cryo-TEM, or in combination with X-ray diffraction or scattering, was used to generate molecular models that showed folding, packing, or orientations of coiled-coils [27,29]. Solution small-angle X-ray scattering (SAXS) is also a powerful technique to build structural models. Comparative structure models are generated from the well-established sequence-to-structure relationships of coiled-coils, followed by fitting to experimental SAXS data. Structural details of the CCPO polygon [30] or polyhedrons [33], including orientation of coiled-coils, were obtained by generating structural modeling and SAXS fitting.

Overall, the principles of modular design strategies used for a variety of self-assembled nanostructures are summarized as follows: Variation of coiled-coils are required in strategic ways that (1) introduce enough connection contacts and that (2) control orientation of coiled-coil interfaces to direct self-assembly into target geometries. In the following sections, I will highlight recent advances of coiled-coil-based nanostructures in three different categories based on their length scales and structural features: (1) unbounded nanostructures, (2) discrete nanoparticles, and (3) well-defined origami nanostructures. Details of building blocks and applied design strategies are summarized in Table 1.

## 3. Supramolecular Nanostructures Built from Coiled-Coils

### 3.1. Unbounded Nanostructures

As discussed in the section of the modular design principles, the control over coiled-coil orientations is essential for programming connections between building blocks into desired geometries. Depending on orientational controls, a significantly large number of building blocks can be connected to unbounded structures. The examples include inter-connected networks in the form of hydrogels [46,47], one-dimensional bundles that grow into fibers [27,31] and tubes [28], and ordered arrays at the nanoscale [39].

**Figure 3:**
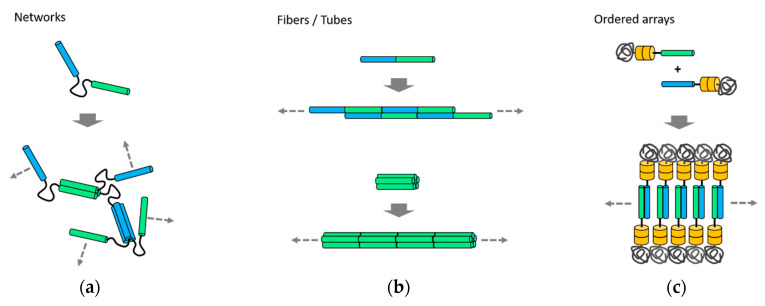
Self-assembly of unbounded nanostructures. (**a**) Formation of networks using coiled-coil fusion proteins with a flexible linker [46,47]; (**b**) assembly and co-directional alignment of coiled-coil building blocks, followed by longitudinal growth into fibers [26,27] or tubes [28]; (**c**) lamellar-ordered arrays assembled from protein-polymer conjugates [39]. Directions of the unbounded growth are indicated by arrows.

Inter-connected networks were built from coiled-coil fragments linked by a soluble and flexible polypeptide midblock [46,47]. It is a polyelectrolyte artificial peptide segment, which was flanked by coiled-coils that oligomerized and mediated reversible physical crosslinking (Figure 3a). The inter-connected networks appeared to be sheet-like nanostructures in the hydrogels [40]. The physical crosslinking can be controlled in response to changes in temperature or pH [46,47], denaturing agents [47], or even electric current [50], which influences thermal stabilities and self-assembling properties of the hydrogels. Programming the mechanical properties was also achieved by changing molecular interactions between the network-forming coiled-coil fusion proteins. For example, erosion rates of hydrogels were tuned by engineering the affinity, oligomerization state, and orientation of the coiled-coil domains [51]. Also, the addition of covalent crosslinking to the protein networks led to time-dependent responses of the hydrogels to mechanical deformation [52]. Single-site mutations in the coiled-coil sequences further programmed the dynamic behavior of the protein network [53]. Such programmable mechanical properties provide opportunities to improve toughness and fracture resistance [52].

Unbounded one-dimensional nanostructures were assembled by the co-directional alignment of coiled-coils (Figure 3b). The Woolfson group first designed coiled-coil fibers by placing heterodimeric coiled-coils in a staggered conformation [26]. In the design, two coiled-coil helices form a dimer that has unbound sticky ends, not blunt. Thus, assembly through the sticky ends aligned the combined coiled-coil interfaces co-directionally, followed by promoting longitudinal growth. These fiber-forming heterodimeric coiled-coils were further modified by rational mutations that introduced charged residues to the outer surfaces, which thickened the fibers [27]. Transmission and electron microscopy and x-ray fiber diffraction revealed hexagonal packing with a periodicity of the length of a single coiled-coil. Coiled-coils were packed along the nanometer-thick (>50 nm) and micrometer-long (>10 µm) fibers. The fiber-forming coiled-coils were also redesigned to create hydrogels [48]. The charged amino acid residues at the characteristic positions *b*, *c*, or *f* (described in Figure 1a) were replaced with the residues with weaker interactions, such as alanine or glutamine, resulting in flexible bundles of thinner and interconnected fibers. Regarding the stability of these fibers, a helix-to-sheet transformation in the structures at temperatures between 20 and 40 °C was recently reported [54]. 

Coiled-coils with blunt ends were also redesigned to form one-dimensional nanostructures (Figure 3b). Complementary charges were introduced to the N- and C-termini of homotrimeric or homotetrameric coiled-coils and the charged blunt ends promoted longitudinal assembly into helical bundles [28]. In contrast to the assembly through the sticky ends, the weak charge interactions at the blunt ends were sufficient for fiber assembly. Using this approach, pentameric, hexameric, and heptameric coiled-coil barrels were redesigned for assembly of nanotubes [28,49]. In the designs, symmetries of the coiled-coils were critical for their packing into ordered tubular suprastructures. Similarly, hollow tubes with diameters ranging from a few hundred nanometers to a micron were assembled from blunt-ended coiled-coils [41]. An isoleucine-rich trimeric coiled-coil was designed by mutations of a natural dimeric coiled-coil GCN4. The coiled-coil was arranged into a hexagonal packing that was stacked and closed into a long hollow suprastructures. In a different approach, heptameric coiled-coils with terminal structures in a lock-washer shape were designed [29]. The shape-complementary charge interactions mediated the end-to-end association of the heptamers that elongated into nanotubes.

Protein arrays with orders at the nanometer scale were fabricated using building blocks containing coiled-coils. While conjugated hybrids of proteins and poly(N-isopropylacrylamide) (PNIPAM) have been developed to assemble ordered nanostructures [55,56], properties of the conjugated proteins were critical to achieving strong ordering [57]. To improve the ordering quality, heterodimeric coiled-coils were fused to a weakly ordering protein, followed by conjugation to PNIPAM (Figure 3c) [39]. Protein arrays assembled from the coiled-coil protein–polymer conjugates displayed lamellar or hexagonal ordered phases, and an improvement in ordering quality was achieved. The nanostructured protein arrays showed enhancement in performance as a biosensor. In a different strategy, ordered arrays in the form of three-dimensional crystals were assembled using coiled-coils modified with metal-ion-binding ligands [58]. Assembly of hexagonal arrays in discs or rods were mediated by binding interactions between metal ions and the conjugated ligands. Site-specific incorporation of guest molecules into the coiled-coil-ordered crystals was also demonstrated.

### 3.2. Discrete Nanoparticles

**Figure 4:**
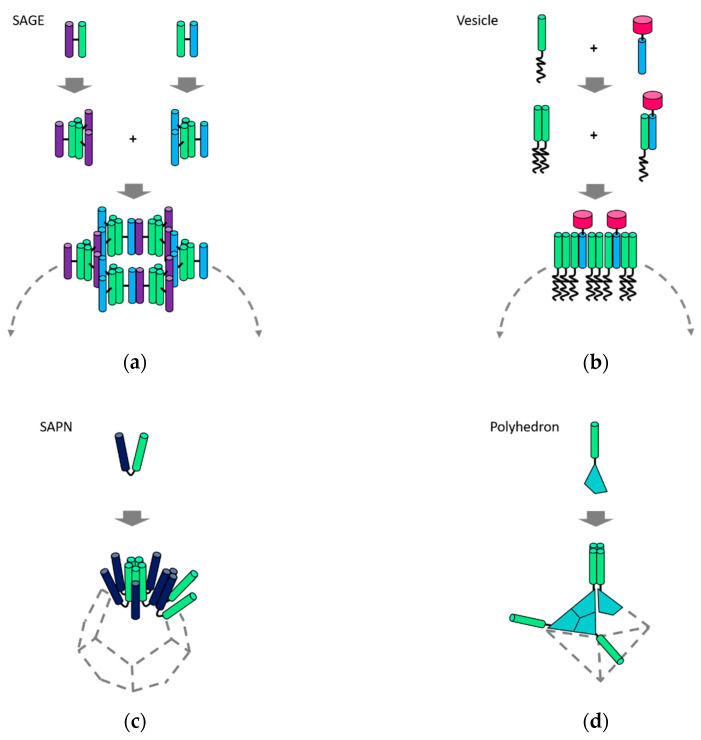
Self-assembly of discrete nanoparticles. (**a**) self-assembled cage-like particles (SAGEs) [35] and (**b**) vesicles [38] are the hollow spheres into which curved lamellar of laterally assembled coiled-coil building blocks are closed; (**c**) a dodecahedron built from the self-assembling peptide nanoparticle (SAPN) system [36] and (**d**) octahedron designed by fusion of coiled-coils to symmetric oligomeric protein folds [37]. These polyhedral nanoparticles are formed by the symmetry-directed assembly.

When the relative orientation of coiled-coil interfaces in a designed protein building blocks is controlled to be within a specific range, closure of coiled-coil assemblies into discrete nanoparticles can be achieved (Figure 2). In such a system, called the self-assembled cage-like particles (SAGEs), curved membrane-like structures were assembled by laterally aligned coiled-coils (Figure 4a) [35]. The SAGE system used a set of heterodimeric and homotrimeric coiled-coils that were conjugated via disulfide bonds. The strategic bridging conjugation controlled the angle between the adjacent coiled-coils. Two bridged complementary building blocks were present as trimeric complexes, which formed unilamellar structures with a curvature when mixed. The curved lamellar became closed into spherical particles with a diameter of around 100 nm [35]. The location of the disulfide bridges was in the middle of the outer surface of coiled-coils, but slightly close to the C-termini. Influenced by a positive charge, the angle between the bridged coiled-coil interfaces was around 33.9° ± 17.2°, according to molecular dynamics simulations. The combination of trimeric and dimeric coiled-coils formed hexagonal networks (Figure 4a), evidenced by characterization using the lateral molecular-force microscopy [35]. Further structural characterization of charge-modified SAGEs after SAGE-templated silicification revealed the presence of both hexagonal and irregular networks on the surface of SiO_2_-SAGEs [59]. Modeling results also indicated that hexagonally arranged packing is not the most stable form [60].

Decoration of SAGEs for applications was achieved by genetic fusion with various peptides or natural proteins. For a controlled delivery of SAGEs into cells, the homotrimeric coiled-coil hub was extended with short charged peptides that enabled alteration of cellular internalization [61]. More functionalities were added to SAGEs by modular decoration with various globular proteins [42]. Green fluorescent protein (GFP), monomeric Cherry fluorescent protein (mCherry), maltose-binding protein, and a luciferase enzyme, were fused to the N- or C-termini of the trimeric coiled-coil hub. In a similar design, an antigen epitope was incorporated into SAGEs, which showed enhanced immunogenicity as protein nanoparticle vaccine delivery [62].

Hollow protein spheres that resemble vesicles were created by genetic fusion of coiled-coils to other types of protein motifs [38]. A heterodimeric coiled-coil Z_E_:Z_R_ [12] (a colon denotes coiled-coil dimerization) were fused to either an elastin-like polypeptide (ELP) or globular protein domains, like mCherry or enhanced GFP (Figure 4b). ELPs are intrinsically disordered peptides that undergo an inverse phase transition from soluble to insoluble conformations, which served as a temperature-responsive hydrophobic block. Globular domains and coiled-coils are hydrophilic and folded into globular or rod shapes, which controlled geometries of the fusion proteins and modulated molecular packing. Since Z_R_ also forms a homodimer with a weaker binding affinity, non-equimolar mixtures of the fusion proteins resulted in rod-coil and globule-rod-coil amphiphiles. Triggered by the inverse phase transition of the ELP domain, hollow protein vesicles were assembled. An empty cavity enclosed by the laterally assembled protein amphiphiles was characterized using confocal microscopy as well as SEM [38]. The curvature of the vesicular membrane was precisely controlled by the relative ratio of the rod-coil and globule-rod-coil protein complexes. Small-angle neutron scattering revealed that the vesicle is unilamellar with a thickness of ~10 nm, and more experimental conditions were explored for a transition to bilamellar structures [43]. 

Polyhedral nanoparticles were created by aligning coiled-coil interfaces into symmetric geometries (Figure 4c). In a system, termed self-assembling peptide nanoparticle (SAPN), pentameric and trimeric coiled-coils were linked to one another by a double-glycine linker [36,63]. An intramolecular disulfide bridge near the linker specified their relative orientations into dodecahedral or icosahedral geometry. This system has been further improved by decoration with various antigen epitopes for the development of protein nanoparticle vaccines. The SAPN vaccines were capable of displaying repetitive epitopes [64,65] or eliminating the need for adjuvants [66]. Versatility of the SAPN-based vaccines was demonstrated against influenza [67], HIV [68,69], and malaria [66,70]. 

The alignment of coiled-coil interfaces into symmetric geometry was also achieved by the genetic fusion of coiled-coils to symmetric oligomeric protein folds (Figure 4d). A C_3_-symmetric trimeric protein esterase was fused to a tetrameric coiled-coil that directed assembly of well-defined octahedral nanostructure [37]. Short flexible linkers with optimized lengths of three or four amino acids were placed between coiled-coils and esterase. Besides, tetrahedral [71] or icosahedral nanostructures [44] were assembled by fusion of the symmetric esterase to trimeric or pentameric coiled-coils.

### 3.3. Origami Nanostructures

The concept of macromolecular origami to create well-defined nanostructures was first realized using DNA. The DNA origami, which refers to a method that folds and assembles DNA fragments into nanostructures, has shown a stunning success in the fabrication of nanoarchitectures in highly customizable shapes and dimensions [72]. The exceptional designability and robustness of the DNA origami are based on the straightforward and highly specific association of complementary DNA fragments. Like DNA, coiled-coils present relatively well-established sequence-to-structure relationships as well as designable interaction specificity and affinity. It is distinct from the characteristics of other protein motifs or domains, which are involved in complicated and cooperative protein–protein interactions in general. These unique characteristics of coiled-coils have been exploited toward straightforward programming of well-defined nanostructure assembly, which is termed CCPO [73].

**Figure 5:**
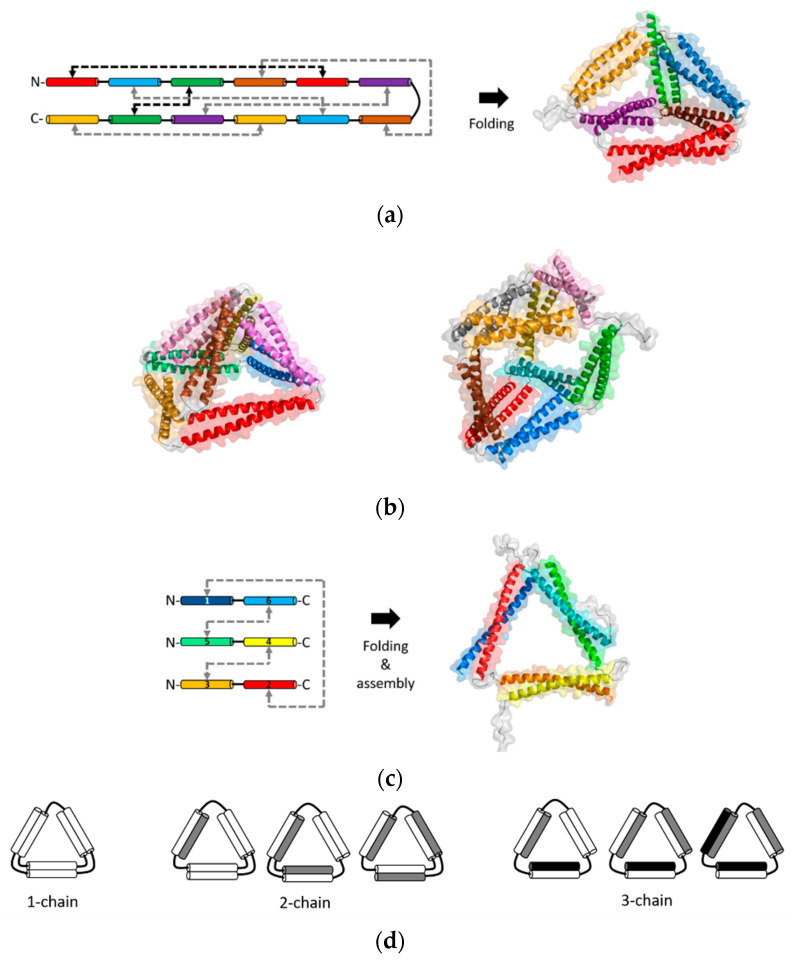
The coiled-coil protein origami (CCPO) nanostructures. (**a**) A single-chain CCPO fusion protein construct (left) and the structural model of the folded tetrahedron (right) [73]; parallel (gray) and antiparallel (black) coiled-coil dimerization interactions are indicated by the arrows; (**b**) structural models of the four-sided pyramid tetrahedron (left) and triangular prism (right) designed by the single-chain CCPO [73]; (**c**) the multi-chain CCPO fusion protein constructs (left) and structural models of the assembled triangle (right) [30]; (**d**) possible triangular topologies are composed of a single chain, two chains, and three chains, respectively. A three-chain topology was chosen for the design of a triangular nanostructure in panel (**c**). The structural models in panels (**a**), (**b**), and (**c**) were from best fits to the SAXS data [30,33].

The methods of CCPO are based on the rationale that orthogonal interactions of individual coiled-coils can be modularly combined for programming interactions of recombinant fusion proteins (Figure 5). Systematic ordering of coiled-coils within a fusion protein chain controls the topology and folding/assembly pathways of desired, well-defined nanostructures. The first-generation of CCPO nanostructures was developed by the Jerala group [32]. This method is based on connecting orthogonal dimeric coiled-coils into a single-chain polypeptide. Coiled-coils were arranged in a topology of a polyhedron, which was folded by orthogonal dimerization of individual coiled-coil pairs guided folding a polyhedron. In particular, a single-chain polypeptide that comprised 12 concatenated coiled-coils was folded into a tetrahedron [32]. A short and non-structured flexible linker was placed between hetero- and homodimeric coiled-coils. Upon folding, the coiled-coil pairs formed six different rod-like edges that were linked into a tetrahedron with a cavity at the core.

Refolding the tetrahedron, the first design of CCPO nanostructure, was slow at nanomolar protein concentrations due to a low solubility [32]. This issue limits CCPO processes under physiological conditions. For this reason, orthogonal coiled-coil modules were modified to be supercharged. The rationale was that modifications to decrease coiled-coil stability, without compromised binding specificity, could avoid possible misfolding [33]. The residues at the positions *b*, *c*, and *f* (illustrated in Figure 1a) can change coiled-coil stability while the orthogonal affinity of a coiled-coil set is not affected [23]. Negatively charged residues were introduced at these positions to decrease the stability of coiled-coils. Using the supercharged coiled-coil modules, a single-chain coiled-coil fusion protein that comprised parallel and antiparallel modules was designed into a tetrahedral topology (Figure 5a) [33]. The tetrahedrons were expressed in bacteria and produced as soluble proteins. Folding of the tetrahedron was also achieved in mammalian cells or mice, demonstrating biocompatibility as well as a strong potential for drug delivery and vaccine applications.

In the single-chain CCPO, all dimeric coiled-coils should be arranged systematically to connect coiled-coil edges in vertices that are linked into a polyhedral structure. An overview of the design principles of the single-chain CCPO is provided in a recent review [73]. Briefly, once a polyhedral target structure is selected, a double Eulerian path is constructed. A linear chain is created from this circular path by selecting an optimal segment order with a low total contact order (TCO) from possible circular permutations. TCO is a scoring parameter that reflects the average distance between paring segments, and a lower TCO increases the design success rate. Next, orthogonal coiled-coils are linked into a polypeptide chain, according to the selected segment order. These steps for a single-chain CCPO design was integrated into a computational design platform, CoCoPOD (https://github.com/NIC-SBI/CC_protein_origami) [33]. This platform can be used to design a CCPO cage structure as well as to build an atomistic structure model. For example, a rectangular pyramid and triangular prism nanostructures were constructed using CoCoPOD (Figure 5b) [33]. A single-chain fusion protein linking six parallel and two antiparallel coiled-coils pairs (16 modules) were designed and folded into a four-sided pyramid. A triangular prism was also designed by constructing a chain that contained six parallel and three antiparallel coiled-coil pairs (18 modules). These two CCPO nanostructures were expressed in bacteria and produced as soluble proteins.

A different CCPO method based on multiple chains was developed by the Keating group and co-workers recently [30]. This method is used to design multiple chains of fusion proteins that link orthogonally interacting coiled-coils. While the single-chain CCPO method constrains multiple coiled-coil interactions within a single-chain, this multi-chain CCPO method arranges coiled-coils to design and program precise interchain interactions. This approach was used to program assembly of a triangular nanostructure, which was achieved by designing three protein chains out of coiled-coils (Figure 5c) [30]. Computationally designed synthetic heterodimeric coiled-coils, named SYNZIPs [16,17], were linked into three separate fusion proteins. When the three linked-SYNZIP fusion proteins were mixed and annealed, orthogonal dimerization of the SYNZIP pairs mediated assembly of a heterotrimeric, triangular protein nanostructure. The programmed association of the linked-SYNZIPs was confirmed by gel electrophoresis in native conditions, followed by secondary structure analysis using circular dichroism spectroscopy. Structural features at the nanometer scale or the atomic level were characterized using the AFM and SAXS, respectively. In a similar way, triangular and square shapes were designed using fusion proteins that linked a single-type heterodimeric coiled-coil [31]. To avoid repeated assembly into unbounded structures such as fibers or bulk phases, the length of linkers between linked coiled-coils was varied for limiting the angle range between coiled-coil edges, resulting in combining 3 or 4 subunits (Figure 3).

The multi-chain CCPO is relatively straightforward as well as flexible for the design and production of protein nanostructures. First, topologies for a target nanostructure can be simply generated by linking fragments that form rod-like coiled-coil edges. Two chains are placed on each edge with a consideration of a range of possible ways to link the edges. For example, topologies that contain one, two, or three chains were possible to create a triangular nanostructure (Figure 5d). For the selected three-chain topology, SYNZIP modules were arranged into the selected topology, followed by choosing a candidate design out of a total of 16 possible designs [30]. The selection of a promising design candidate was made by eliminating design candidates containing building blocks that might form undesired competing structures such as homodimers or heterodimers of linked-SYNZIP subunits. Second, such undesired competing byproducts can be reduced by adjusting annealing protocols or removed by size-exclusion chromatography (SEC). In the design of a triangle, the formation of a homodimer of linked SYNZIPs was reduced by changing the molar ratio of the subunits or changing the cooling rate during thermal annealing. After annealing, triangles were separated from mixtures of undesired byproducts by SEC [30]. 

The use of multiple chains for a CCPO design provides great potential towards improved design capabilities. While a single-chain CCPO topology requires to satisfy the double Eulerian path [73], the use of multiple chains for CCPO avoids such constraints and enables the generation of various possible topologies (Figure 5d). This advantage can potentially improve the designability toward more complex CCPO nanostructures than polyhedrons or polygons. However, some essential improvements need to be made. First, the angles at each vertex should be precisely controlled to assemble desired shapes. For example, the shape of polygonal nanostructures composed of more than three edges can be distorted without using angle-controlling linkers. Conformational constraints by placing disulfide bonds [36] or short linkers showed promises [31]. Also, engineering rigid linkers with well-defined angles will improve the designability. Second, more sets of orthogonally interacting coiled-coils are needed. More fundamental knowledge and datasets on coiled-coil specificity and stability will enable the de novo design of larger sets of orthogonal coiled-coils. Lastly, fundamentals on cooperative interactions between many chains as well as controllable folding pathways will enable the effective design of complex topologies using many coiled-coil chains.

## 4. Conclusions and Future Directions

In this review, I have discussed the strategies to use coiled-coils as modules that assemble a variety of protein nanostructures with distinct structural features, which include networks, fibers, tubes, ordered arrays, cages, vesicles, polyhedrons, and polygons. The advances in coiled-coil protein nanostructures have shown great promise for the fabrication of biocompatible nanomaterials with tunable structural features. I expect that the modular and rational design enabled by coiled-coils will play a critical role in the development of more complex, well-defined, and functional nanomaterials.

A factor to consider for further improvement of nanostructure design is stability. Many of the nanostructures discussed in this review were stable, confirmed by structure characterizations or tolerance to genetic fusion with larger protein domains. The CCPO polyhedrons and triangle were stable for at least weeks at 4 °C, which was similar to the stability of natural proteins [30,33]. The cages [42], vesicles [38], or polyhedrons [45] tolerated several proteins fused to the coiled-coils within the assemblies. Nonetheless, the robustness of coiled-coils can be attenuated by variation of coiled-coils, depending on how they are modified or engineered for nanostructure assembly. For example, when connected to other protein components, even well-designed stable coiled-coils had unexpected changes in the oligomerization state [74]. Also, a helix-to-sheet structural transformation of the fiber-forming coiled-coil peptides was observed unanticipatedly [54]. In applications, such information on the stability of nanostructures is critical. Therefore, more studies on parameters that can affect the stability of coiled-coils within the assembled nanostructures are needed. It will provide the foundation for the development of applications.

While biodegradability and biocompatibility of protein materials hold potential for biomedical applications [75], functionality is also likely to be an essential factor to consider. An immediate challenge is to improve the design capability to control material properties as well as functionalities toward specific applications. For example, structural features at the nanometer scale can be engineered to modulate molecular transport [76], biocatalysis [77,78], ligand–receptor interactions [79], biomechanics [80], and cellular internalization [61] or localization [81]. To date, the modular addition of functionalities to coiled-coil nanostructures have been achieved by the genetic fusion of functional protein domains [38,42,82]. Beyond this, correlations between nanoscale features, functional efficacy, and structural stability will be desired for the systematic design of functional protein nanomaterials using coiled-coils.

## Figures and Tables

**Table 1:** Representative protein nanostructures assembled from coiled-coil-based building blocks.

Category	Type	Length Scale ^1^	Design Strategy	References
Unbounded nanostructures	Network	~500 nm ~50–100 nm	Coiled-coil fusion Modification	[46,47,48]
	Fiber	~50–100 nm	Coiled-coil fusion/Modification	[26,27]
	Tube	~3–100 nm	Modification	[28,49]
	Ordered array	~25 nm	Fusion to other domains	[39]
Discrete nanoparticles	Cage	~100 nm	Coiled-coil conjugation	[35]
	Vesicle	~1–2 µm	Fusion to other domains	[38]
	Polyhedron	~16–24 nm~18–25 nm	Coiled-coil fusion Fusion to other domains	[36,37,44]
Origami nanostructures	Polyhedron	~5–10 nm	Single-chain CCPO	[32,33]
	Polygon	~5–10 nm	Multi-chain CCPO	[30,31]

^1^ The length scale indicates the characteristic dimension of each protein nanostructure: the thickness of networks, fibers or tubes, the domain spacing of ordered arrays, and the diameter of cages, vesicles, polyhedrons, or polygons.

## References

[1] Lupas A.N., Bassler J. (2017). Coiled coils–a model system for the 21st century. Trends Biochem. Sci..

[2] Hai T., Curran T. (1991). Cross-family dimerization of transcription factors Fos/Jun and ATF/CREB alters DNA binding specificity. Proc. Natl. Acad. Sci. USA.

[3] Reinke A.W., Baek J., Ashenberg O., Keating A.E. (2013). Networks of bZIP protein-protein interactions diversified over a billion years of evolution. Science.

[4] Whitby F.G., Phillips G.N. (2000). Crystal structure of tropomyosin at 7 Ångstroms resolution. Proteins Struct. Funct. Bioinform..

[5] Koubassova N., Bershitsky S., Tsaturyan A. (2018). Effects of an Interchain Disulfide Bond on Tropomyosin Structure: A Molecular Dynamics Study. Int. J. Mol. Sci..

[6] Mason J.M., Arndt K.M. (2004). Coiled coil domains: Stability, specificity, and biological implications. Chembiochem.

[7] Lupas A.N., Gruber M. (2005). The structure of α-helical coiled coils. Adv. Protein Chem..

[8] Woolfson D.N., Bartlett G.J., Bruning M., Thomson A.R. (2012). New currency for old rope: From coiled-coil assemblies to α-helical barrels. Curr. Opin. Struct. Biol..

[9] Apostolovic B., Danial M., Klok H.-A. (2010). Coiled coils: Attractive protein folding motifs for the fabrication of self-assembled, responsive and bioactive materials. Chem. Soc. Rev..

[10] Grigoryan G., Keating A.E. (2008). Structural specificity in coiled-coil interactions. Curr. Opin. Struct. Biol..

[11] Wood C.W., Woolfson D.N. (2018). C CB uilder 2.0: Powerful and accessible coiled-coil modeling. Protein Sci..

[12] Moll J.R., Ruvinov S.B., Pastan I., Vinson C. (2001). Designed heterodimerizing leucine zippers with a ranger of pIs and stabilities up to 10−15 M. Protein Sci..

[13] Gradišar H., Jerala R. (2011). De novo design of orthogonal peptide pairs forming parallel coiled-coil heterodimers. J. Pept. Sci..

[14] Fletcher J.M., Boyle A.L., Bruning M., Bartlett G.J., Vincent T.L., Zaccai N.R., Armstrong C.T., Bromley E.H.C., Booth P.J., Brady R.L. (2012). A basis set of de novo coiled-coil peptide oligomers for rational protein design and synthetic biology. ACS Synth. Biol..

[15] Grigoryan G., Reinke A.W., Keating A.E. (2009). Design of protein-interaction specificity gives selective bZIP-binding peptides. Nature.

[16] Reinke A.W., Grant R.A., Keating A.E. (2010). A synthetic coiled-coil interactome provides heterospecific modules for molecular engineering. J. Am. Chem. Soc..

[17] Thompson K.E., Bashor C.J., Lim W.A., Keating A.E. (2012). SYNZIP protein interaction toolbox: In vitro and in vivo specifications of heterospecific coiled-coil interaction domains. ACS Synth. Biol..

[18] Negron C., Keating A.E. (2014). A set of computationally designed orthogonal antiparallel homodimers that expands the synthetic coiled-coil toolkit. J. Am. Chem. Soc..

[19] Crooks R.O., Baxter D., Panek A.S., Lubben A.T., Mason J.M. (2016). Deriving heterospecific self-assembling protein–protein interactions using a computational interactome screen. J. Mol. Biol..

[20] Crooks R.O., Lathbridge A., Panek A.S., Mason J.M. (2017). Computational Prediction and Design for Creating Iteratively Larger Heterospecific Coiled Coil Sets. Biochemistry.

[21] Thomson A.R., Wood C.W., Burton A.J., Bartlett G.J., Sessions R.B., Brady R.L., Woolfson D.N. (2014). Computational design of water-soluble α-helical barrels. Science.

[22] Kaplan J.B., Reinke A.W., Keating A.E. (2014). Increasing the affinity of selective bZIP-binding peptides through surface residue redesign. Protein Sci..

[23] Drobnak I., Gradišar H., Ljubetič A., Merljak E., Jerala R. (2017). Modulation of coiled-coil dimer stability through surface residues while preserving pairing specificity. J. Am. Chem. Soc..

[24] Robson Marsden H., Kros A. (2010). Self-assembly of coiled coils in synthetic biology: Inspiration and progress. Angew. Chem. Int. Ed..

[25] Wu Y., Collier J.H. (2017). α-Helical coiled-coil peptide materials for biomedical applications. Wiley Interdiscip. Rev. Nanomed. Nanobiotechnol..

[26] Pandya M.J., Spooner G.M., Sunde M., Thorpe J.R., Rodger A., Woolfson D.N. (2000). Sticky-end assembly of a designed peptide fiber provides insight into protein fibrillogenesis. Biochemistry.

[27] Papapostolou D., Smith A.M., Atkins E.D.T., Oliver S.J., Ryadnov M.G., Serpell L.C., Woolfson D.N. (2007). Engineering nanoscale order into a designed protein fiber. Proc. Natl. Acad. Sci. USA.

[28] Burgess N.C., Sharp T.H., Thomas F., Wood C.W., Thomson A.R., Zaccai N.R., Brady R.L., Serpell L.C., Woolfson D.N. (2015). Modular design of self-assembling peptide-based nanotubes. J. Am. Chem. Soc..

[29] Xu C., Liu R., Mehta A.K., Guerrero-Ferreira R.C., Wright E.R., Dunin-Horkawicz S., Morris K., Serpell L.C., Zuo X., Wall J.S. (2013). Rational design of helical nanotubes from self-assembly of coiled-coil lock washers. J. Am. Chem. Soc..

[30] Park W.M., Bedewy M., Berggren K.K., Keating A.E. (2017). Modular assembly of a protein nanotriangle using orthogonally interacting coiled coils. Sci. Rep..

[31] Boyle A.L., Bromley E.H.C., Bartlett G.J., Sessions R.B., Sharp T.H., Williams C.L., Curmi P.M.G., Forde N.R., Linke H., Woolfson D.N. (2012). Squaring the circle in peptide assembly: From fibers to discrete nanostructures by de novo design. J. Am. Chem. Soc..

[32] Gradišar H., Božič S., Doles T., Vengust D., Hafner-Bratkovič I., Mertelj A., Webb B., Šali A., Klavžar S., Jerala R. (2013). Design of a single-chain polypeptide tetrahedron assembled from coiled-coil segments. Nat. Chem. Biol..

[33] Ljubetič A., Lapenta F., Gradišar H., Drobnak I., Aupič J., Strmšek Ž., Lainšček D., Hafner-Bratkovič I., Majerle A., Krivec N. (2017). Design of coiled-coil protein-origami cages that self-assemble in vitro and in vivo. Nat. Biotechnol..

[34] Yeates T.O., Liu Y., Laniado J. (2016). The design of symmetric protein nanomaterials comes of age in theory and practice. Curr. Opin. Struct. Biol..

[35] Fletcher J.M., Harniman R.L., Barnes F.R.H., Boyle A.L., Collins A., Mantell J., Sharp T.H., Antognozzi M., Booth P.J., Linden N. (2013). Self-assembling cages from coiled-coil peptide modules. Science.

[36] Raman S., Machaidze G., Lustig A., Aebi U., Burkhard P. (2006). Structure-based design of peptides that self-assemble into regular polyhedral nanoparticles. Nanomed. Nanotechnol. Biol. Med..

[37] Sciore A., Su M., Koldewey P., Eschweiler J.D., Diffley K.A., Linhares B.M., Ruotolo B.T., Bardwell J.C.A., Skiniotis G., Marsh E.N.G. (2016). Flexible, symmetry-directed approach to assembling protein cages. Proc. Natl. Acad. Sci. USA.

[38] Park W.M., Champion J.A. (2014). Thermally triggered self-assembly of folded proteins into vesicles. J. Am. Chem. Soc..

[39] Paloni J.M., Olsen B.D. (2020). Coiled-Coil Domains for Self-Assembly and Sensitivity Enhancement of Protein–Polymer Conjugate Biosensors. ACS Appl. Polym. Mater..

[40] Xu C., Kopeček J. (2008). Genetically engineered block copolymers: Influence of the length and structure of the coiled-coil blocks on hydrogel self-assembly. Pharm. Res..

[41] Nambiar M., Nepal M., Chmielewski J. (2019). Self-Assembling Coiled-Coil Peptide Nanotubes with Biomolecular Cargo Encapsulation. ACS Biomater. Sci. Eng..

[42] Ross J.F., Bridges A., Fletcher J.M., Shoemark D., Alibhai D., Bray H.E.V., Beesley J.L., Dawson W.M., Hodgson L.R., Mantell J. (2017). Decorating Self-Assembled Peptide Cages with Proteins. ACS Nano.

[43] Jang Y., Choi W.T., Heller W.T., Ke Z., Wright E.R., Champion J.A. (2017). Engineering globular protein vesicles through tunable self-assembly of recombinant fusion proteins. Small.

[44] Cristie-David A.S., Chen J., Nowak D.B., Bondy A.L., Sun K., Park S.I., Banaszak Holl M.M., Su M., Marsh E.N.G. (2019). Coiled-coil-mediated assembly of an icosahedral protein cage with extremely high thermal and chemical stability. J. Am. Chem. Soc..

[45] Cristie-David A.S., Koldewey P., Meinen B.A., Bardwell J.C.A., Marsh E.N.G. (2018). Elaborating a coiled-coil-assembled octahedral protein cage with additional protein domains. Protein Sci..

[46] Petka W.A., Harden J.L., McGrath K.P., Wirtz D., Tirrell D.A. (1998). Reversible hydrogels from self-assembling artificial proteins. Science.

[47] Xu C., Breedveld V., Kopeček J. (2005). Reversible Hydrogels from Self-Assembling Genetically Engineered Protein Block Copolymers. Biomacromolecules.

[48] Banwell E.F., Abelardo E.S., Adams D.J., Birchall M.A., Corrigan A., Donald A.M., Kirkland M., Serpell L.C., Butler M.F., Woolfson D.N. (2009). Rational design and application of responsive α-helical peptide hydrogels. Nat. Mater..

[49] Thomas F., Burgess N.C., Thomson A.R., Woolfson D.N. (2016). Controlling the assembly of coiled–coil peptide nanotubes. Angew. Chem. Int. Ed..

[50] Lin Y., An B., Bagheri M., Wang Q., Harden J.L., Kaplan D.L. (2017). Electrochemically Directed Assembly of Designer Coiled-Coil Telechelic Proteins. ACS Biomater. Sci. Eng..

[51] Shen W., Zhang K., Kornfield J.A., Tirrell D.A. (2006). Tuning the erosion rate of artificial protein hydrogels through control of network topology. Nat. Mater..

[52] Dooling L.J., Buck M.E., Zhang W.-B., Tirrell D.A. (2016). Programming Molecular Association and Viscoelastic Behavior in Protein Networks. Adv. Mater..

[53] Dooling L.J., Tirrell D.A. (2016). Engineering the Dynamic Properties of Protein Networks through Sequence Variation. ACS Cent. Sci..

[54] Roberts E.K., Wong K.M., Lee E.J., Le M.M., Patel D.M., Paravastu A.K. (2018). Post-assembly α-helix to β-sheet structural transformation within SAF-p1/p2a peptide nanofibers. Soft Matter.

[55] Thomas C.S., Glassman M.J., Olsen B.D. (2011). Solid-state nanostructured materials from self-assembly of a globular protein–polymer diblock copolymer. ACS Nano.

[56] Dong X., Obermeyer A.C., Olsen B.D. (2017). Three-Dimensional Ordered Antibody Arrays Through Self-Assembly of Antibody–Polymer Conjugates. Angew. Chem. Int. Ed..

[57] Huang A., Paloni J.M., Wang A., Obermeyer A.C., Sureka H.V., Yao H., Olsen B.D. (2019). Predicting Protein–Polymer Block Copolymer Self-Assembly from Protein Properties. Biomacromolecules.

[58] Nepal M., Sheedlo M.J., Das C., Chmielewski J. (2016). Accessing three-dimensional crystals with incorporated guests through metal-directed coiled-coil peptide assembly. J. Am. Chem. Soc..

[59] Galloway J.M., Senior L., Fletcher J.M., Beesley J.L., Hodgson L.R., Harniman R.L., Mantell J.M., Coombs J., Rhys G.G., Xue W.-F. (2017). Bioinspired Silicification Reveals Structural Detail in Self-Assembled Peptide Cages. ACS Nano.

[60] Mosayebi M., Shoemark D.K., Fletcher J.M., Sessions R.B., Linden N., Woolfson D.N., Liverpool T.B. (2017). Beyond icosahedral symmetry in packings of proteins in spherical shells. Proc. Natl. Acad. Sci. USA.

[61] Beesley J.L., Baum H.E., Hodgson L.R., Verkade P., Banting G.S., Woolfson D.N. (2018). Modifying self-assembled peptide cages to control internalization into mammalian cells. Nano Lett..

[62] Morris C., Glennie S.J., Lam H.S., Baum H.E., Kandage D., Williams N.A., Morgan D.J., Woolfson D.N., Davidson A.D. (2019). A Modular Vaccine Platform Combining Self-Assembled Peptide Cages and Immunogenic Peptides. Adv. Funct. Mater..

[63] Indelicato G., Wahome N., Ringler P., Müller S.A., Nieh M.-P., Burkhard P., Twarock R. (2016). Principles governing the self-assembly of coiled-coil protein nanoparticles. Biophys. J..

[64] Schroeder U., Graff A., Buchmeier S., Rigler P., Silvan U., Tropel D., Jockusch B.M., Aebi U., Burkhard P., Schoenenberger C.-A. (2009). Peptide nanoparticles serve as a powerful platform for the immunogenic display of poorly antigenic actin determinants. J. Mol. Biol..

[65] IYang Y., Ringler P., Müller S.A., Burkhard P. (2012). Optimizing the refolding conditions of self-assembling polypeptide nanoparticles that serve as repetitive antigen display systems. J. Struct. Biol..

[66] Kaba S.A., Brando C., Guo Q., Mittelholzer C., Raman S., Tropel D., Aebi U., Burkhard P., Lanar D.E. (2009). A nonadjuvanted polypeptide nanoparticle vaccine confers long-lasting protection against rodent malaria. J. Immunol..

[67] Karch C.P., Li J., Kulangara C., Paulillo S.M., Raman S.K., Emadi S., Tan A., Helal Z.H., Fan Q., Khan M.I. (2017). Vaccination with self-adjuvanted protein nanoparticles provides protection against lethal influenza challenge. Nanomed. Nanotechnol. Biol. Med..

[68] Karch C.P., Bai H., Torres O.B., Tucker C.A., Michael N.L., Matyas G.R., Rolland M., Burkhard P., Beck Z. (2019). Design and characterization of a self-assembling protein nanoparticle displaying HIV-1 Env V1V2 loop in a native-like trimeric conformation as vaccine antigen. Nanomed. Nanotechnol. Biol. Med..

[69] Wahome N., Pfeiffer T., Ambiel I., Yang Y., Keppler O.T., Bosch V., Burkhard P. (2012). Conformation-specific display of 4E10 and 2F5 epitopes on self-assembling protein nanoparticles as a potential HIV vaccine. Chem. Biol. Drug Des..

[70] Guo Q., Dasgupta D., Doll T.A.P.F., Burkhard P., Lanar D.E. (2013). Expression, purification and refolding of a self-assembling protein nanoparticle (SAPN) malaria vaccine. Methods.

[71] Badieyan S., Sciore A., Eschweiler J.D., Koldewey P., Cristie-David A.S., Ruotolo B.T., Bardwell J.C.A., Su M., Marsh E.N.G. (2017). Symmetry-directed self-assembly of a tetrahedral protein cage mediated by de novo-designed coiled coils. ChemBioChem.

[72] Rothemund P.W.K. (2006). Folding DNA to create nanoscale shapes and patterns. Nature.

[73] Lapenta F., Aupič J., Strmšek Ž., Jerala R. (2018). Coiled coil protein origami: From modular design principles towards biotechnological applications. Chem. Soc. Rev..

[74] Cristie-David A.S., Sciore A., Badieyan S., Escheweiler J.D., Koldewey P., Bardwell J.C.A., Ruotolo B.T., Marsh E.N.G. (2017). Evaluation of de novo-designed coiled coils as off-the-shelf components for protein assembly. Mol. Syst. Des. Eng..

[75] DeFrates K., Markiewicz T., Gallo P., Rack A., Weyhmiller A., Jarmusik B., Hu X. (2018). Protein polymer-based nanoparticles: Fabrication and medical applications. Int. J. Mol. Sci..

[76] Park W.M., Champion J.A. (2013). Two-Step Protein Self-Assembly in the Extracellular Matrix. Angew. Chem. Int. Ed..

[77] Park W.M., Yee C.M., Champion J.A. (2016). Self-assembled hybrid supraparticles that proteolytically degrade tumor necrosis factor-α. J. Mater. Chem. B.

[78] Caparco A.A., Bommarius B.R., Bommarius A.S., Champion J.A. (2020). Protein-inorganic calcium-phosphate supraparticles as a robust platform for enzyme co-immobilization. Biotechnol. Bioeng..

[79] Simnick A.J., Valencia C.A., Liu R., Chilkoti A. (2010). Morphing low-affinity ligands into high-avidity nanoparticles by thermally triggered self-assembly of a genetically encoded polymer. ACS Nano.

[80] Diehl M.R., Zhang K., Lee H.J., Tirrell D.A. (2006). Engineering cooperativity in biomotor-protein assemblies. Science.

[81] Shemesh O.A., Linghu C., Piatkevich K.D., Goodwin D., Gritton H., Romano M.F., Siciliano C.A., Gao R., Yu C.-C.J., Tseng H. (2019). Precision calcium imaging of dense neural populations via a cell body-targeted calcium indicator. bioRxiv.

[82] Park W.M., Champion J.A. (2016). Colloidal Assembly of Hierarchically Structured Porous Supraparticles from Flower-Shaped Protein–Inorganic Hybrid Nanoparticles. ACS Nano.

